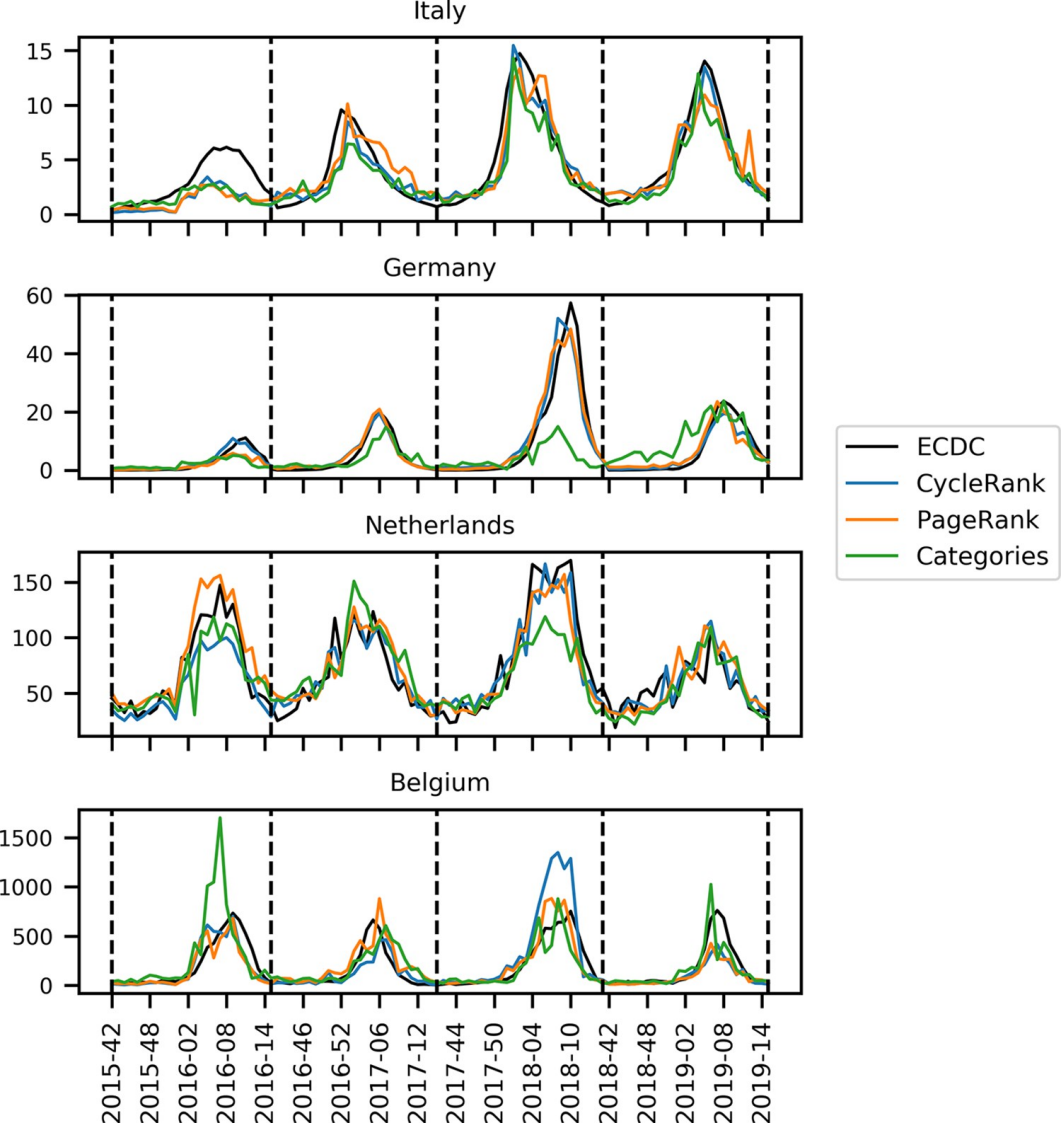# Correction: A general method for estimating the prevalence of influenza-like-symptoms with Wikipedia data

**DOI:** 10.1371/journal.pone.0314302

**Published:** 2024-11-18

**Authors:** Giovanni De Toni, Cristian Consonni, Alberto Montresor

In Table 1, the headings of columns three (Dutch) and four (German) are swapped. Please see the correct Table 1 here.

**Table 1:** Selected Wikipedia categories. We first selected the Italian categories, and then we chose the corresponding German and Dutch translations. We report the English categories for reference.

English	Italian	Dutch	German
Viral diseases	Malattie infettive virali	Virusziekte	Virale Infektionskrankheit
Infectious diseases	Malattie infettive	Infectieziekte	Infektionskrankheit
Epidemics	Epidemie	Epidemie	Epidemie
Viruses	Virus	Virus	Viren, Viroide und Prionen
Vaccines	Vaccini	Vaccin	Impfstoff
Medical signs	Segni clinici	Symptoom	Krankheitssymptom

In Figs 1 and 2, the labels of the x-axis are incorrect. The data show the evolution of ILI and the prediction of the model for four flu seasons, but the axis labels refer to only two seasons. Please see the correct Fig 1 and Fig 2 here.

**Fig 1:**
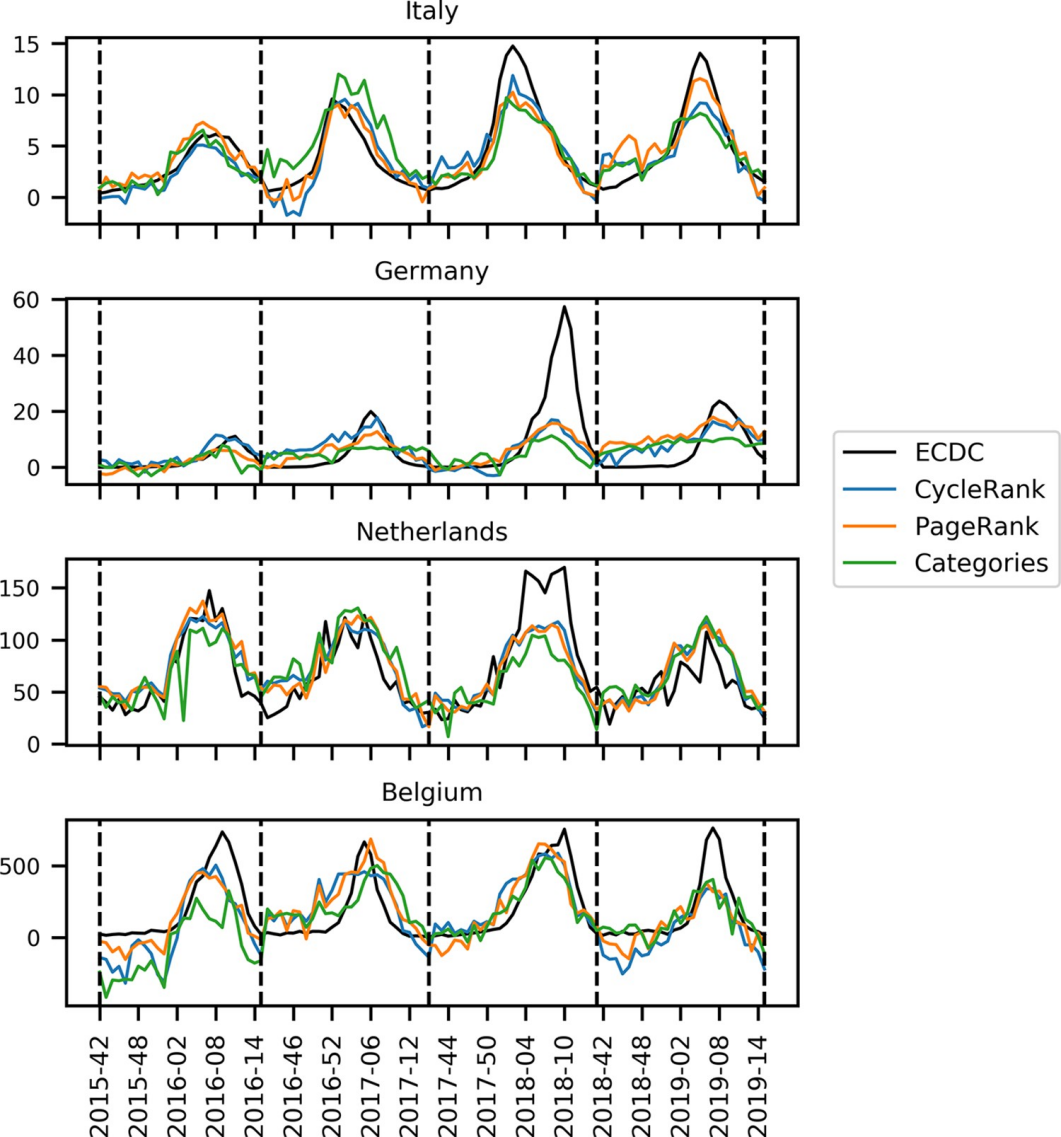


**Fig 2:**