# Supplementary material for: A general method for estimating the prevalence of influenza-like-symptoms with Wikipedia data
Source: PLoS One. 2021 Aug 31;16(8):e0256858. doi: 10.1371/journal.pone.0256858 (PMC8407583; doi:10.1371/journal.pone.0256858)
Supplement: Attachment — Submitted filename: Response_to_Reviewers.pdf [file pone.0256858.s001.pdf]

Response letter for paper # PONE-D-20-37326
A general method for estimating the prevalence of Influenza-Like-Symptoms with Wikipedia data

The authors would like to thank the anonymous reviewers for their time and effort to provide extensive reviews and invaluable feedback. This document lists the changes that we have made in response to the reviewers' comments.

In the paper, the major changes have been highlighted in yellow/strikethrough. We also have improved the text and corrected typos in several places, as suggested, without highlighting.

Giovanni De Toni
Cristian Consonni
Alberto Montresor

Reviewer #1:

The present manuscript presents a methodology to nowcast influenza based on Wikipedia page visits. The method is interesting, the subject timely, and has the potential to be broadly applied to different languages. Using human curation to pre-select features is also a potentially relevant approach. However, the manuscript as presented has some concerning issues, discussed below. Moreover, the authors' bold claim in the discussion that they "have shown the feasibility of using automatic methods to extract relevant predictors ..." has no support. None of the feature-selection methods are new and the model performance is poor. Below is a point by point discussion of the paper.

Major points
R1.1 Nowcasting: The model is intended to perform nowcasting, which typically implies training and testing models on past data to monitor ongoing outbreaks. However, the authors are testing the model against whole seasons where the data is standardized (at least the Wikipedia dataset is - line 82). How could this be achieved without having the full data of the season? How well would the model(s) perform in a scenario of "true" nowcasting (ie, predict the next week(s) based on the last few weeks)?

A1.1 *The standardization is performed separately for the train and test set. Namely, we compute the mean and standard deviation of the train set, and we use them to standardize singularly each week of the test set. Obviously, here we are assuming that our train and test sets are coming from a similar underlying generation process. Therefore, there is no information shared between the train and test sets.*
Our models perform nowcasting given the pageviews computed for the target week without taking into account the previous ones. This was a methodological choice, since we wanted to try to predict influenza levels based solely on the page views of a given week, without using any additional information.
We updated the corresponding sections in the paper to better explain these points.

R1.2 Quality of the models: The prediction of the peak week is more interesting from the point of view of nowcasting (although concerns about standardization remain). However, judging it in relation to the ground truth is only one of the relevant metrics. Nowadays

medical systems make predictions about peaks based on epidemiological data and models. How better is your model in relation to these more traditional approaches?
Unfortunately the models behave very poorly against the ground truth (table 4). This raises the question of how useful are they. Also, the Categories model misses a peak altogether in Germany (2nd panel of fig.1) and this should be discussed.

A1.2 *To address this issue, we have added to our work an additional generalized linear model taken from other relevant papers (e.g., McIver et al., 2014) which should serve as an additional comparison against the simple Lasso linear model.*

R1.3 Differences between tools: The interpretation of the differences between the two datasets PV and PC+PV is not straightforward. The authors discuss it in terms of having more or less data, concluding that, for cycle rank and categories, more data is better while for pagerank it is worse. There is not enough evidence to say this because PC+PV not only has more data but it has different data (ex: PC includes bot visits). Also, there is no obviously better method or dataset, with them methods working differently in different countries. This raises concerns regarding generabillity.

A1.3 *We have updated the sections in the paper to address this concern by adding new experiments and by using more sophisticated preprocessing techniques on the PV and PV+PC dataset. See Table 3 and Table 4 for the updated evaluation. The new results show with more clarity that there are indeed benefits when using more data to train the models (even if they are collected from a different source).*

R1.4 Comparison to previous works: there have been many other efforts of nowcasting using Google searches, Wikipedia visits, on-call phone systems, self-reported symptom apps, etc. The authors should discuss their findings and model in comparison to previous published work and how their approach improves current thinking.

A1.4 *Other recent and state-of-the-art approaches generally come without disclosing the code used to train their models or without the dataset employed. For example, Google searches or other corporate data are not generally available to external researchers or the public. Therefore, this limits our ability to produce a fair comparison between our methods and the ones presented in the relevant literature. Moreover, even if the techniques are detailed precisely, the software used is usually proprietary, thus creating a challenge from a reproducibility standpoint. To at least provide some additional context, we updated the introduction of this work by adding some recent works to address also current methodologies and state-of-the-art methods.*

R1.5 Minor points Page 2, starting at line 66 - It would be good to include some of Wikipedia's methodology to arrive at these numbers since provenance of visits is important for the results of the paper.

A1.5 *The section 2 was updated to add an intuition about how the provenance counts are computed. For a more complete overview, we refer to the original source, Wikistats[1], for the complete description of the methodology used.*

R1.6 Why was performance measured only with mean Pearson correlation? I would like to see both R^2 and Mean squared error as well.

A1.6 *In the revised version of our paper, we added the R^2 score. We did not report the MSE since the various incidence datasets had different distributions and numerosity, thus the relative MSE change between the various models could have been misleading. Therefore, we deem the MSE to add more confusion to the overall picture rather than additional insights.*

R1.7 In section 6, feature analysis. It is unclear why the authors focus only on pages positively correlated with cases. Negative correlations can be just as useful (albeit more difficult to interpret).

A1.7 *We updated section 6.1 to address this concern. We argue that negative weights can be interpreted as a balancing factor for the positive weights assigned to certain features such as to provide better estimates which minimize the model's MSE.*

R1.8 The paper has multiple typos. Here is a non-exhaustive list:
Legend of table 1 should be "... German and Dutch translations..."
line 121 "...in order to avoid making unnecessary..."
line 129 till the end of that paragraph. Replace Wikipedia's pages with Wikipedia pages line 160/161 "...circular walks that start and end..."
line 348 "...require conducting..."

A1.8 *We have corrected the typos listed here and we have performed a detailed proofreading of the paper.*

R1.9 Finally, the quality of the figures is very poor but we assume it will be improved before publication.

A1.9 *We inserted in the revised manuscript a new version of the pictures. Their quality should be better now.*

---

1 https://stats.wikimedia.org/wikimedia/squids/SquidReportPageViewsPerLanguageBreakdown.htm

Reviewer #2:

This paper describes a work aimed at applying a machine learning model on Wikipedia's page views of a selected group of articles to obtain accurate estimates of influenza-like illnesses incidence in four European countries: Italy, Germany, Belgium, and the Netherlands. This effort falls in a decade-long research line aimed at using non-traditional data sources generated by digital platforms to track and now-cast the Influenza-like Illness circulation among the general population. The goal is to extend the already existing studies in the USA context to a broader Europe-focussed one. It is a very interesting attempt that, on the other hand, has still some issues. Some comments are reported in the following:

R2.1 In the introduction, only a little of the decade-long efforts and scientific studies to nowcast and forecast influenza through digital data (not necessarily with machine learning) are mentioned.

A2.1 *We updated the introduction of this work by adding some recent works to address also current methodologies and state-of-the-art methods.*

R2.2 How do they avoid overfitting and using features that are highly correlated? Feature selection is performed by the machine learning approach but still for each model tens of features are used. Did the authors check for collinearity?

A2.2 *Regularization techniques are used to ensure feature selection and to prevent overfitting. Namely, LASSO and Elastic-Net penalties push the models to give positive weights to pages which are highly correlated with the influenza incidence. Moreover, the penalties select a set of features which present little collinearity, since we want to maximize the amount of information given by each of them (e.g., if two features are correlated, then the penalties will push the model towards choosing just one of them, since they carry the same information).*

R2.3 Can this approach be used for other countries/languages? Wikipedia provides the country from which the pageviews are generated, so even for pages in English or French, it is possible to disentangle the provenance of the clicks.

A2.3 *We argue that our approach is indeed multilingual and it can be effortlessly applied to other countries/languages. However, we would like to point out that the information about the provenance of visits to Wikipedia articles is not publicly provided, since it is highly sensitive information that would put Wikipedia readers' privacy at risk. To ensure the reproducibility of our work we used only publicly available data. In the case of languages for which the page views provenance is more diverse (e.g., English, Arabic), this could indeed lead to a decrease in accuracy of the models. However, we point out that McIver et Al. 2014 were able to use English pageviews to predict accurately USA incidence without the need for disentangling the provenance of page views.*

R2.4 What is the use in public health, given the not-so-accurate estimate of the peak? Would a different regression model help on this?

A2.4 *Despite the peak prediction not being completely satisfactory, we argue that being able to know the influenza incidence with a reasonable accuracy for a given week is still an asset. These estimates can already be provided without the need for any additional process to be put in place for doctors and health professional laboratory tests or any further action from the public. This includes also people who do not interact with their local health system at all. Moreover, these data could give an overview of the epidemiological situation of a country in a matter of seconds. As we noted in the conclusion of our work, the purpose of these methods is to complement more traditional human activities, and we underline that no matter how sophisticated our model can be or how much data we possess, there are still common pitfalls and limitations that should be taken into account. These risks have already been out in the past, for example (Lazer et al., 2014) tackled the limitations of Google Flu Trend project. Our aim with this paper is to provide a fully reproducible work, because the data, the methods and the code are available for the community without any restriction.*

R2.5 Would the integration of these data with other digital data or traditional surveillance data help in making a more accurate estimate of the peak?

A2.5 *Yes, see the paper by (Santillana et al., 2015) and (Perrotta et al., 2017).*

References

McIver DJ, Brownstein JS (2014) Wikipedia Usage Estimates Prevalence of Influenza-Like Illness in the United States in Near Real-Time. PLOS Computational Biology 10(4): e1003581. https://doi.org/10.1371/journal.pcbi.1003581

Daniela Perrotta, Michele Tizzoni, and Daniela Paolotti. 2017. Using Participatory Web-based Surveillance Data to Improve Seasonal Influenza Forecasting in Italy. In *Proceedings of the 26th International Conference on World Wide Web* (*WWW '17*). International World Wide Web Conferences Steering Committee, Republic and Canton of Geneva, CHE, 303–310. DOI:https://doi.org/10.1145/3038912.3052670

Santillana M, Nguyen AT, Dredze M, Paul MJ, Nsoesie EO, et al. (2015) Combining Search, Social Media, and Traditional Data Sources to Improve Influenza Surveillance. PLOS Computational Biology 11(10): e1004513. https://doi.org/10.1371/journal.pcbi.1004513
