# Supplementary material for: A general method for estimating the prevalence of influenza-like-symptoms with Wikipedia data
Source: PLoS One. 2021 Aug 31;16(8):e0256858. doi: 10.1371/journal.pone.0256858 (PMC8407583; doi:10.1371/journal.pone.0256858)
Supplement: Attachment — Submitted filename: Response_to_Reviewers_v2.pdf [file pone.0256858.s002.pdf]

Response letter for paper # PONE-D-20-37326
A general method for estimating the prevalence of Influenza-Like-Symptoms with Wikipedia data

The authors would like to thank the anonymous reviewers for their time and effort to provide these additional reviews and helpful feedback. This document lists the changes that we have made in response to the reviewer's comments.

In the paper, the major changes have been highlighted in yellow/strikethrough. We also have improved the text and corrected additional typos in several places, as suggested, without highlighting.

Giovanni De Toni
Cristian Consonni
Alberto Montresor

Reviewer #1:

The authors did a thorough job in addressing the concerns and the manuscript is significantly improved as a result. Particularly the addition of the GLM model helps justify the bolder claims.

Major points:

R1.1 I am afraid we were not clear in our previous comments. The medical authorities in each country typically use compartmental models based on SIR, that rely on information from previous weeks (and/or seasons). The question is whether using Wikipedia data to predict influenza is better than these traditional models. We understand this may be beyond the scope of the paper but would like to see it mentioned in the discussion. The GLM model visibly improves predictions and was a good addition to the paper.

A1.1 *It is true that we do not provide a direct comparison between compartmental methods and our data-driven approach. We updated the "Discussion" session of the paper to mention this issue as a possible future extension. Our methodology is intended to be an addition to these standard techniques to support and diversify the sources from which we try to predict how the influenza trend will evolve.*

R1.2 the discussion of PV versus PV+PC is now more complete but did not fully address our concerns. The fact that PV and PC data are different, limits the discussion of "more vs. better". For example, what would the results look like if only PC data was used? This is no longer a key claim of the paper, but we would still argue that the authors have not consistently shown that using more data improves the models.

A1.2 *We updated the experiments in the paper by showing also the Pearson Correlation Coefficients of models trained by using only the PC dataset. From the results, it can be seen that models trained with the PC data underperform with respect to models trained with PV or PV+PC. Moreover, from a practical perspective, the PC dataset has been discontinued by*

Wikipedia and models trained with it may fall prey to distribution shift (since the collection methodology changed) or other phenomena which could make them less precise in the future.

R1.3 The discussion of previous work is now more complete. As a small note, we are not sure what the authors mean about Google searches not being available to researchers. It is true that the underlying method/algorithm is not known, but normalized data is: https://trends.google.com/trends/

A1.3 *We thank the reviewer for having pointed this out. We would like to expand on this and explain the practical reason why we did not consider Google data for this work:*

1. *Even if those data are available to the public, Google might change their availability or restrict access, so scientists could not build models that can last over the years. We are aware that Wikipedia is not exempt from these issues in principle - in the sense that the project may somehow die in the long term - but we still think it offers better guarantees in terms of openness.*

2. *Using Wikipedia data, we can better control and understand the features we are dealing with. Google provides only the normalized trend, but we have no control over any intermediate processing and the collection process.*

3. *By incorporating Google data, we might defeat the entire purpose of understanding the extent of Wikipedia's ability to predict the influenza trend.*

On the other hand, in the case of production-ready systems, it might be advisable to develop an ensemble of multiple models that can leverage different data sources to improve the quality of the predictions.
