# Supplementary material for: A general method for estimating the prevalence of influenza-like-symptoms with Wikipedia data
Source: PLoS One. 2021 Aug 31;16(8):e0256858. doi: 10.1371/journal.pone.0256858 (PMC8407583; doi:10.1371/journal.pone.0256858)
Supplement: Attachment — Submitted filename: Response_to_Reviewers_v3.pdf [file pone.0256858.s003.pdf]

**Response letter for paper # PONE-D-20-37326**
A general method for estimating the prevalence of Influenza-Like-Symptoms with Wikipedia data

We would like to thank the anonymous reviewer for their time and effort in providing these additional reviews and helpful feedback. As we indicated in the submission system, we would like these revisions to be public, as we have found the process to be well-conducted and the feedback to be very beneficial to the quality of the paper. This document lists the changes that we have made in response to the reviewer's comments.

We added the additional comments regarding Google Flu Trends in the Discussion section, and we also worked to improve the manuscript's readability.

In the version, we highlighted significant changes in blue/strikethrough. We have also improved the text and corrected additional typos in several places, as suggested, without highlighting.

Giovanni De Toni
Cristian Consonni
Alberto Montresor
